# Rab27a Is Required for Human Cytomegalovirus Assembly

**DOI:** 10.1371/journal.pone.0015318

**Published:** 2010-12-08

**Authors:** Alberto Fraile-Ramos, Victoria Cepeda, Edo Elstak, Peter van der Sluijs

**Affiliations:** 1 Department of Molecular and Cell Biology, Centro Nacional de Biotecnología, Consejo Superior de Investigaciones Científicas, Madrid, Spain; 2 Department of Cell Biology, University Medical Center Utrecht, Utrecht, The Netherlands; University of Geveva, Switzerland

## Abstract

Human cytomegalovirus (HCMV) completes its final envelopment on intracellular membranes before it is released from the cell. The mechanisms underlying these processes are not understood. Here we studied the role of Rab27a, a regulator of lysosome-related organelle transport, in HCMV production. HCMV infection increased Rab27a expression, and recruitment of Rab27a to membranous strutures at the assembly site. Immuno-gold labelling demonstrated association of Rab27a with viral envelopes. CMV production was reduced after knock-down of Rab27a, and in Rab27a-deficient *ashen* melanocytes. This study shows a requirement for Rab27a in the CMV life cycle and suggests that CMV and LRO biogenesis share common molecular mechanisms.

## Introduction

Human cytomegalovirus (HCMV), a member of the *Betaherpesvirinae* subfamily, is a widespread pathogen in man that causes an asymptomatic and often times latent infection. However, HCMV can also be life threatening for immunocompromised patients such as individuals with HIV, organ transplant recipients and conatally infected neonates [1]. HCMV consists of a capsid containing the double-stranded DNA genome surrounded by the tegument and a lipid envelope with embedded glycoproteins. It is thought that nucleocapsids assembled within the infected cell nucleus are released into the cytoplasm through the nuclear membrane. In the cytoplasm, capsids acquire a set of tegument proteins and undergo a final envelopment by wrapping into membranes before they are secreted to the extra-cellular space [2]. While the nature of the membrane that HCMV uses for its envelope has been controversial for some time [3], [4], [5], [6], [7], [8], [9], our recent results show that final envelopment occurs into a hybrid compartment or in transport vesicles between the trans-Golgi network (TGN) and endosomes [10], the mechanism of which remains to be defined.

HCMV increases expression of several *Rab* genes whose products control membrane traffic [11]. One of these is *Rab27a* that regulates secretion of lysosomes-related organelles (LRO) which share features with multivesicular bodies (MVBs)/lysosomes [12]. *Rab27a* mutations cause pigment as well as lytic granule transport defects, accounting for partial albinism and immune disorder in Griscelle syndrome type 2 [13] and the corresponding mouse model *ashen* [14]. The genetic findings together with the functional characterisation of Rab27a cemented a role for this GTPase in secretion of LRO such as melanosomes in pigmented cells [15] and lytic granules in cytotoxic T lymphocytes [13]. In addition, Rab27a is expressed in other specialized secretory cells [16] and participates in insulin granule secretion in pancreatic beta cells [17].

Given its function in LRO release, we investigated a role for Rab27a in HCMV secretion. We report that Rab27a might be required not only in virus transport/secretion but also in infectious viruses production. These data indicate that CMV and LRO biogenesis share common molecular mechanisms.

## Materials and Methods

### Reagents and antibodies

Tissue culture reagents and tissue culture plastic were from LabClinics S.A. (Barcelona, Spain), and chemicals from Sigma Aldrich (Madrid, Spain).

Antibodies were from indicated sources: anti-β-actin AC-15 (Sigma Aldrich); anti-HCMV glycoprotein H [18] and anti-herpes simplex virus type 1 glycoprotein D [19] (Dr H. Browne, University of Cambridge, UK); anti-MCMV IE1 Croma 101 [20] (Dr S. Jonjić, University of Rijeka, Croatia); anti-HCMV pp28 [21] (Dr T. Shenk, Princeton University, NJ, USA); anti GFP (Dr D. Shima, University College London, UK); anti-Rab27a [22]; HCMV antibody-negative and –positive sera from anonymous healthy donors (Comunidad de Madrid Blood Transfusion Centre, Spain) were provided by Dr H. Reyburn (CNB, Madrid, Spain). Fluorescent, HRP- and gold-conjugated secondary antibodies were from Invitrogen S.A. (Barcelona, Spain), Thermo Scientific (Madrid, Spain) and BioCell International (Cardiff, UK) respectively.

### Cells and viruses

Immortalised human foreskin fibroblasts (BJ1) cells were from Clontech (California, USA), mouse *melan-a* and *ashen*-3 melanocytes from the Wellcome Trust Functional Genomics Cell Bank (London, UK), and MeWo cells and MEFs were kind gifts of Drs L. Montoliu (CNB, Madrid, Spain) and A. Angulo (IDIBAPS, Barcelona, Spain) respectively. Cells were maintained as recommended by suppliers. BJ1 and MeWo cells were transduced with viral vectors expressing shRNAs. Stable BJ1 and MeWo cell lines were selected in medium containing 10 and 2 µg/ml puromycin, respectively, and Rab27a silencing was analysed by Western blot. BJ1 YFP-Rab27a expressing cells were sorted with an ALTRA HyPerSort flow cytometer (Beckman Coulter, Inc., Palo Alto, USA). HCMV Towne and RCMV288 strains were propagated and titered on BJ1 cells [10]. MCMV Smith strain was propagated in MEFs and titered by plaque assay. For RNA-interference-mediated gene silencing viral vectors were prepared with pMDG, p8.91 and retroviral expression plasmids encoding non-target control (SHC002) and Rab27a shRNAs: TRCN0000005294, TRCN0000005295, TRCN0000005296, TRCN0000005297 and TRCN0000005298 (Mission® TRC-Hs shRNA libraries, Sigma Aldrich) [23], as described [24]. YFP-Rab27a was extended with Gateway recombination sequences and transferred via pDONR207 (Invitrogen) to the lentiviral plasmid pLNT-SFFV-WPRE-Gateway obtained through Dr G. Griffiths from Dr A. Thrasher (University College London, UK). Recombinant lentivirus was made as described above. All synthetic cDNAs were verified by dye termination sequencing.

### Microscopy methods

BJ1 cells growing on glass coverslips were mock infected, or infected with HCMV Towne strain at a moi of 0.5. At 5 dpi cells were fixed, and processed for immunofluorescence microscopy [10]. Cells were analysed using a Leica DMI6000 microscope equipped with a Leica TCS-SP5 multispectral confocal laser scanning system. To prepare the figures, digital images were transferred to Adobe Photoshop and adjusted so that intensity values extended over the full measurable range (0–255 grey levels).

BJ1 YFP-Rab27a expressing cells were infected with HCMV Towne strain at a moi of 3. At 5 dpi supernatants were harvested for immuno-gold labeling of isolated viruses and cells were fixed and processed for cryo-sectioning and electron microscopy (EM). Sections were labelled with anti-GFP and 10 nm gold-coupled secondary antibodies in the presence of 10% human serum [4]. Sections were examined with a transmission electron microscope (model JEOL 1011). To prepare the figure, images were recorded onto electron image film (SO-163; Kodak). Negatives were scanned and images adjusted as above. For immuno-gold labelling of isolated viruses, viral suspensions were adsorbed onto Formvar coated EM grids, permeabilised with saponin to access the inner leaflet of the viral envelope and stained with primary antibodies and 10 nm gold-coupled protein-A (EM Lab, Utrecht University, The Netherlands) as previously described [10]. The specificity of the immuno-gold labelling was assayed by omitting the primary antibody, or by using an anti-herpes simplex virus type 1 glycoprotein D antibody as negative control. Samples were examined and images were recorded as above. ∼200 nm in diameter spherical enveloped particles were classified as virions. At least 20 viral particles were analyzed for the control samples, while a minimum of 100 virions were examined and the number of gold particles associated to each virion was calculated.

### HCMV infection in Rab27a shRNA expressing cells

For HCMV infection assays, 2.5×10^5^ untransduced, non-target control and Rab27a shRNA expressing cells were infected with RCMV288 at a moi of 0.5. After 3 days, a portion of the cells was fixed, analyzed by flow cytometry and the number of infected cells was assayed by GFP expression. At 4 dpi, a portion of the cells was lysed and the levels of Rab27a and HCMV proteins were analysed by Western blot. The remainder of the cells was washed and fresh medium was added to collect viruses secreted into the supernatants. At day 5 pi, supernatant and cells were harvested, and extracellular and cell-associated infectious RCMV288 assayed on BJ1 cells as previously described [9]. In these assays 56 hpi, cells were fixed, analysed by flow cytometry and the number of infected cells, i.e. number of infectious virus particles, was assessed by GFP expression.

### MCMV infection in Rab27a-deficient cells

For MCMV infection assays, 2.5×10^5^ *melan-a* and *ashen-3* melanocytes were infected with MCMV Smith strain at a moi of 1. At 24 hpi, cells growing on glass coverslips were fixed and MCMV IE1 protein expression was assayed by immunofluorescence, while the rest of cells were washed and fresh medium was added. After 48 h, extra-cellular and cell-associated infectious viruses were determined by plaque assay.

### Western blotting

For the analysis of Rab27a protein expression during HCMV infection, BJ1 cells growing in 60-mm tissue culture dishes were mock infected or infected with HCMV Towne strain at a moi of 3. At time 0 mock-infected and at 1, 2, 3, 4, and 5 dpi HCMV-infected cells were lysed in 100 µl non-reducing SDS-PAGE sample buffer, separated on 10% SDS-PAA gels and transferred to PVDF membranes. Blots were analyzed as described [10]. Autoradiography films were scanned and the bands were quantified with the use of the public domain ImageJ 1.43 program. Protein loading was normalised to actin expression levels. For the analysis of Rab27a depletion, Mewo cells and HCMV-infected untransduced, non-target control and Rab27a shRNAs-expressing BJ1 cells were lysed and the expression levels of Rab27a were assayed as above. The levels of expression of HCMV proteins were assayed with a HCMV antibody-positive serum since it has been shown that serum from patients with CMV infection reacts with a large number of structural virion proteins [25].

## Results

### Expression of Rab27a in HCMV-infected cells

**Figure 1:**
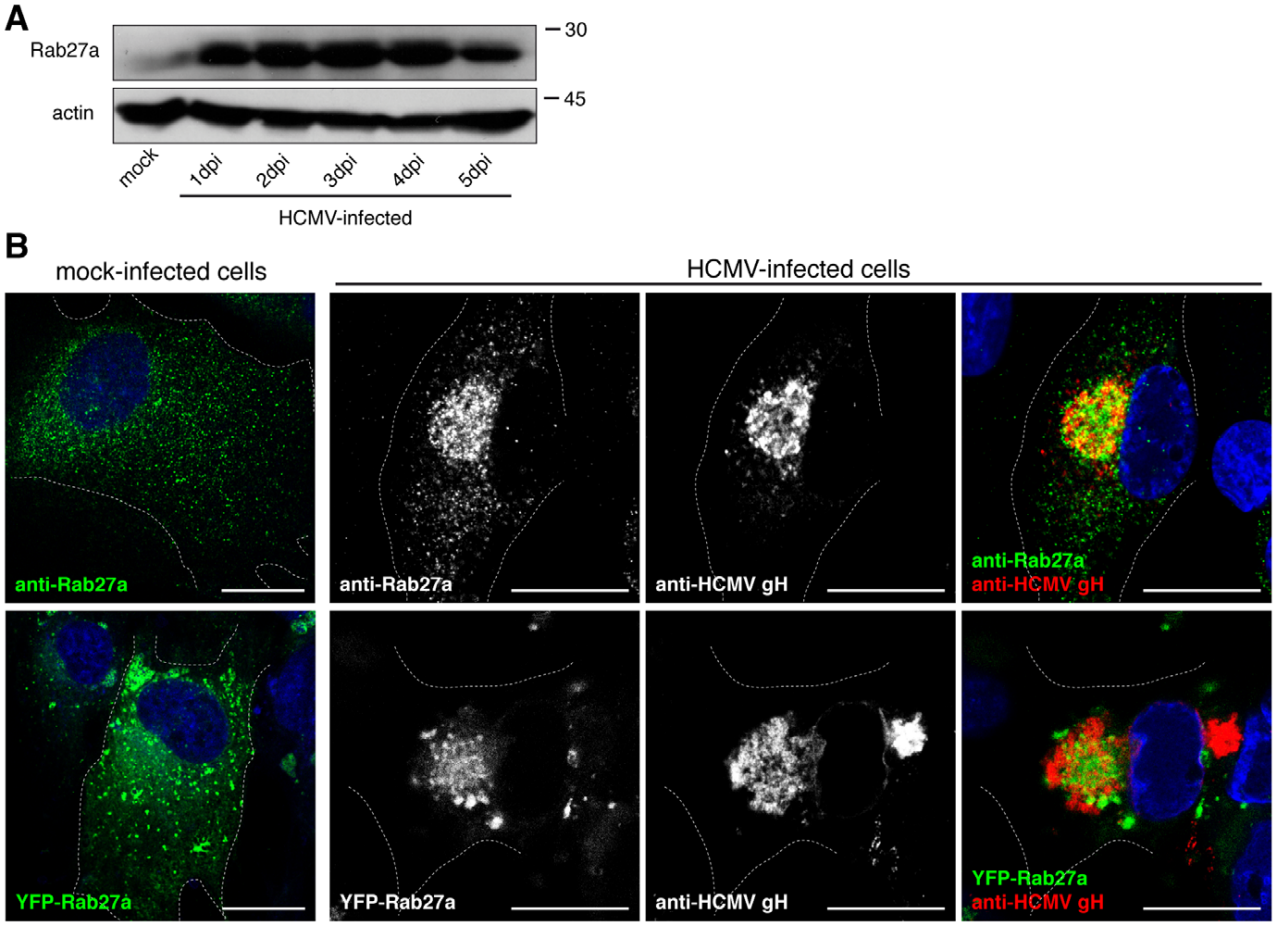
Expression and localisation of Rab27a in HCMV-infected cells. (A) Rab27a protein expression during HCMV infection. Equal number of BJ1 cells mock infected or infected with HCMV at a moi of 3 were lysed at the indicated times. Rab27a was analyzed by Western blot where actin served as loading control Autoradiography films were scanned; images were cropped and assembled with Adobe Photoshop. Molecular weights in kDa are indicated. (B) Subcellular localisation of Rab27a in HCMV-infected cells. BJ1 and BJ1-YFP-Rab27a cells were either mock infected (left panels) or infected with HCMV at a moi of 0.5 (right panels). After 5dpi, cells were fixed, permeabilised and stained with anti-Rab27a (green in upper panels) and anti-HCMV glycoprotein gH (red) antibodies. In BJ1-YFP-Rab27a cells, YFP was directly imaged. DNA was stained with DAPI (blue). Dashed lines show the outline of the cells. Scale bars, 20 µm.

Analysis of host cell gene expression during HCMV infection revealed the up-regulation of Rab27a [11]. To characterise the regulation of Rab27a, BJ1 immortalised human foreskin fibroblast cells were infected with the Towne strain of HCMV and Rab27a protein levels were analysed by Western blot. Within 1 day after HCMV infection, Rab27a expression was increased 3 fold, while after 5 days expression levels started to decrease somewhat (Fig. 1A).

### Localisation of Rab27a in HCMV-infected cells, and isolated HCMV particles

**Figure 2:**
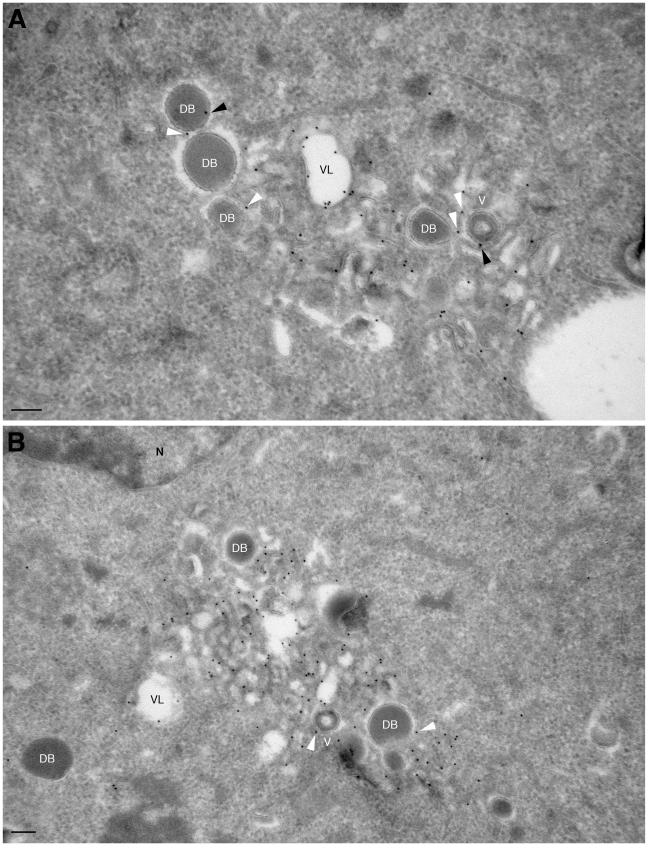
Immuno EM localisation of Rab27a in HCMV-infected cells. (A and B) Cryosections of BJ1 YFP-Rab27a cells HCMV infected for 5 days at a moi of 3 were labelled for YFP with 10 nm gold-conjugated secondary antibodies. Gold particles were seen over the membrane of vacuoles, small vesicles and tubules, as well as the envelope of virions and dense bodies (black arrowhead), and the vacuole containing them (white arrowhead). N, nucleus; VL, vacuole; V, virion; D, dense bodies. Scale bars, 200 nm.

The subcellular localisation of Rab27a was next analysed by immunofluorescence microscopy at 5 dpi when virus assembly areas are established in the juxta-nuclear region, and viral particles are secreted [6]. In BJ1 cells Rab27a was present in many small cytoplasmic punctuated structures (Fig. 1B). In HCMV-infected cells the scattered distribution of Rab27a was changed and Rab27a appeared to be recruited to the assembly site where it partially co-localised with HCMV glycoprotein H (Fig. 1B). To further locate Rab27a we carried out immuno-gold labelling of cryosections followed by electron microscopy (EM). Because the anti-Rab27 serum did not allow us to consistently detect the endogenous protein in cryosections of HCMV-infected cells, we expressed a YFP-tagged Rab27a. In these cells YFP-Rab27a located throughout the cytoplasm (Fig. 1B) while HCMV-infection relocated YFP-Rab27a to the assembly site (Fig. 1B), as observed for endogenous Rab27a immunoreactivity. Some diffuse YFP-Rab27 appeared to partially overlap with a fraction of HCMV gH at the centre of the assembly site, although HCMV gH structures that lacked YFP-Rab27a were always seen (Fig. 1B). These immunofluorescence results suggest that a portion of the two proteins might be associated to the same structures while other may localise to distinct micro-domains of the assembly site. Partial co-localisation could also be due to the accessibility of the antibodies to their antigens, that may differ between cellular and viral membranes as we have previously observed [10]. To further analyse the possible interaction of Rab27a with viral particles we next localised YFP-Rab27a in HCMV-infected cells by immuno-EM. The rabbit antiserum anti-GFP showed low levels of nonspecific binding over the nucleus and mitochondria. We noted cytoplasmic staining as well, but this could represent a pool of cytoplasmically localised YFP-Rab27a that is not in the GTP form. Specific labelling for YFP-Rab27a was seen over the membrane of vacuoles, small vesicles and tubules (Fig. 2), these membranes were in the vicinity of virions and dense bodies (DBs) that underwent final envelopment (Fig. 2). DBs represent globular accumulations of viral tegument proteins with the capacity to acquire an envelope by the same mechanism as virions. Interestingly, YFP-Rab27a was also found associated to the envelope of virus particles and DBs (Fig. 2A), and seen over the vacuole enclosing them (Fig. 2A–B).

**Figure 3:**
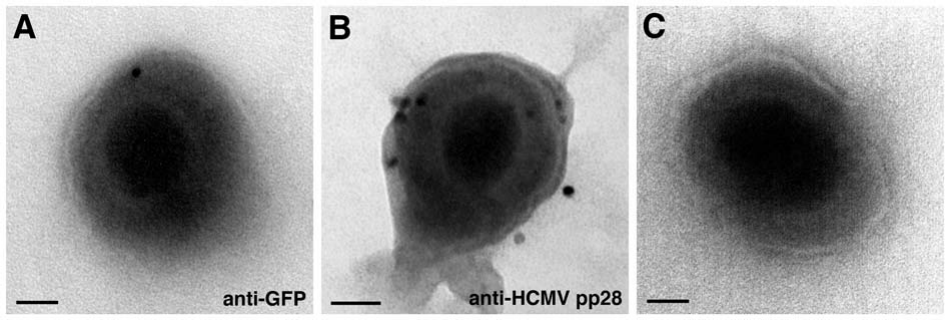
Immuno EM localisation of Rab27a on the viral envelope of isolated HCMV viral particles. Isolated viral particles from BJ1 YFP-Rab27a cells HCMV infected for 5 days were permeabilised with saponin and labelled with antibodies against YFP (A) and HCMV tegument viral protein pp28 (B), and 10 nm protein-A gold. (C) General morphology of HCMV virions by negative staining with 2% uranyl acetate. When viral particles were partially disrupted, uranyl acetate revealed the nucleocapsids. Scale bar, 50 nm.

To analyse the incorporation of YFP-Rab27a into the viral envelope, we subjected isolated virus particles to immuno-gold EM. During the wrapping process membrane-bound cytosolic Rab27a would be incorporated over the interior of the virus particle. Therefore, enveloped particles were permeabilised with saponin prior labelling and as a control for the accessibility to the inner leaflet of the viral envelope, an antibody against the HCMV tegument viral protein pp28 was used (Figure 3B, 94 of 122 virions were labelled with 3.2±1.1 gold particles per virion). The specificity of the labelling was assayed by excluding the primary antibody (in this case just 1 of 25 virions were labelled with one gold particle), or using antibodies against herpes simplex virus type 1 glycoprotein D as negative control (5 of 120 virions were labelled with one gold particle). YFP-Rab27a was detected on 60 of 130 virions with 1.5±0.3 gold particles (Figure 3A).

Together these results indicate that HCMV induces expression of Rab27a which is recruited to the assembly site, and part of the protein is associated to virus-wrapping membranes and incorporated into the viral envelope.

### Requirement of Rab27a for HCMV production

**Figure 4:**
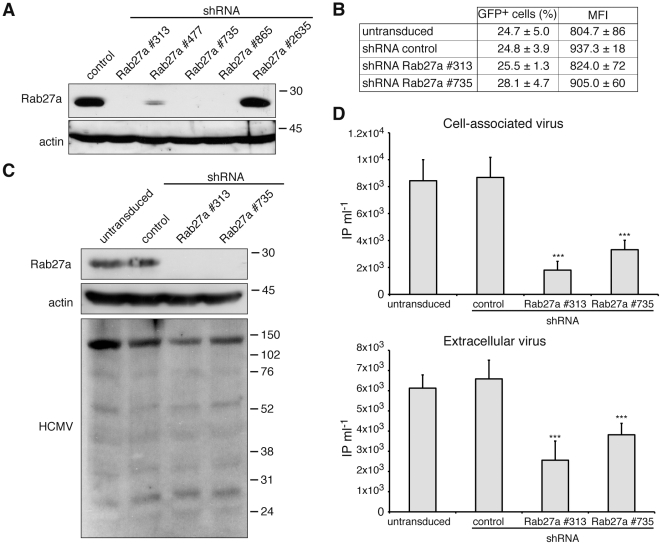
HCMV infection after knock-down of Rab27a. (A) Mewo cells expressing non-target control and Rab27a shRNAs were lysed, and Rab27a expression was determined by Western blot. Actin served as loading control. Molecular weights in kDa are indicated. (B–D) Infectious viruses produced in BJ1 Rab27a shRNAs expressing cells. BJ1 untransduced, non-target control and Rab27a shRNAs expressing cells were infected with RCMV288 at a moi of 0.5. After 3 days a fraction of the cells was analysed by flow cytometry to assess the number of infected cells by GFP expression (B). Some cells were lysed at 4 dpi, and expression of Rab27a, actin and HCMV was analyzed by Western blot (C). At 5 dpi, supernatants and the remainder of the cells were harvested, and the number of extracellular and cell-associated infectious viruses was determine on fresh BJ1 cells as described in Materials and Methods (D). Data are means plus standard deviation (n = 3). ***, *P*<0.001. MFI: mean fluorescence intensity. IP: infectious particles.

To study the function of Rab27a in HCMV transport/secretion we used shRNA. First, we tested Rab27a shRNAs in a human melanoma cell line (MeWo) by Western blot. shRNA Rab27a #313, #735 and #865 completely silenced Rab27a, while shRNA Rab27a #477 yielded a more moderate effect (Fig. 4A). HCMV-infected MeWo cells did not secrete viral particles (data not shown), thus Rab27a #313 and #735 shRNAs were selected to generate BJ1 cells in which Rab27a was stably depleted. To test whether Rab27a is required for viral particle transport/secretion, cells were infected with RCMV288, a recombinant strain of HCMV AD169 expressing GFP under control of an HCMV early promoter [26]. The number of GFP-expressing cells and the mean fluorescence intensity was the same in Rab27a-depleted cultures as in non-target control shRNA-expressing cells suggesting that Rab27a is not involved in HCMV entry and early viral genes expression (Fig. 4B). After 4 dpi some cells were lysed and the expression of Rab27a and HCMV proteins was analysed by Western blot. Rab27a levels were undetectable in HCMV-infected silenced cells (Fig. 4C), as observed in MeWo cells, while HCMV protein levels were not significantly altered in control and Rab27a shRNA-expressing cells, indicating that depletion of Rab27a did not affect the expression of HCMV proteins (Fig. 4C). A blot with HCMV-negative serum was included as control and no reactivity was observed (data not shown). Five dpi, supernatants and cells were harvested, and the production of secreted and cell-associated infectious HCMV was determined. We found a reproducible and significant ∼2-fold reduction in the number of infectious viruses secreted from Rab27a-silenced cells compared with control conditions (*P*<0.001) (Fig. 4D). Unexpectedly, a ∼4-fold reduction was observed when cell-associated viruses from Rab27a silenced cells were assayed (*P*<0.001; Fig. 4D). Together these results showed that Rab27a depletion reduced both the number of cell-associated infectious viruses and virus particle secretion suggesting a role for Rab27a in HCMV production.

### Reduced production of MCMV in *ashen* Rab27a-deficient melanocytes

**Figure 5:**
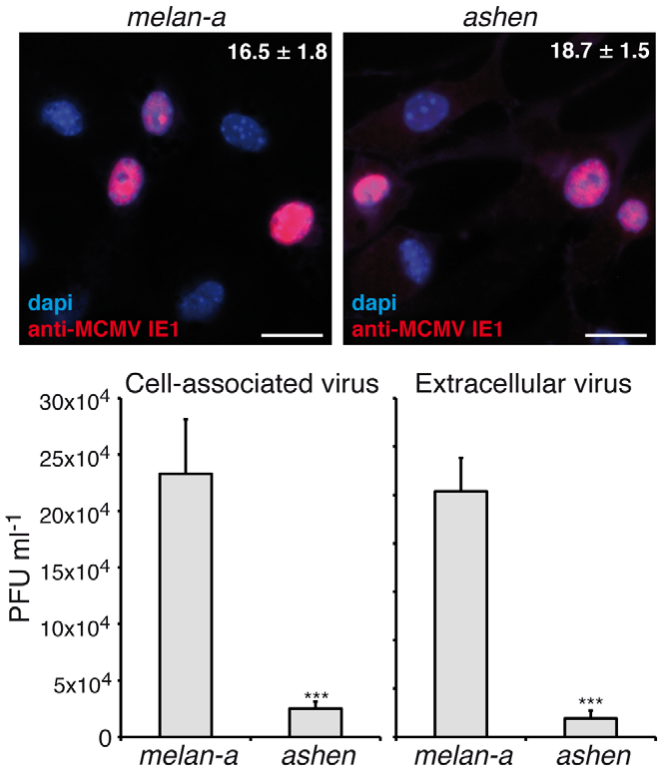
MCMV infection in Rab27a-deficient melanocytes. M*elan-a* and *ashen-3* melanocytes were infected with MCMV, and after 24 h some cells were fixed and MCMV IE1 protein expression analysed by immunofluorescence microscopy (magenta in upper panels). At 48 hpi, supernatants and cells were harvested, and the number of extracellular and cell-associated infectious viruses were determined by plaque assay (histograms). Data are means plus standard deviations (n = 2). ***, P<0.001. PFU: plaque forming units.

Murine cytomegalovirus (MCMV) provides a model for the analysis of replication and dissemination of HCMV [27]. Melanosome transport defects in *ashen* mice are caused by a mutation in the *Rab27a* locus [14]. To confirm and extend the role of Rab27a in infectious virus production, we analysed MCMV yield in melanocytes derived from Rab27a-deficient *ashen* mice. MCMV completes its life cycle faster than HCMV, and viral particles are detected in MCMV-infected melanocytes supernatants from 48 hours post infection (data not shown). *Ashen-3* and control *Melan-a* cells were infected with MCMV, yielding the same fraction of cells expressing MCMV immediate early protein 1 (IE1) (Fig. 5), showing that Rab27a is immaterial for viral entry in several cell types. At 48 hpi, supernatants and cells were harvested, and the production of infectious MCMV was determined. We observed a reproducible and significant ∼8-fold decrease in the number of infectious viruses secreted (*P*<0.001), and ∼10-fold reduction in the cell-associated viruses (*P*<0.001) from *ashen-3* cells compared with *melan-a* cells (Fig. 5). These results showed that lack of functional Rab27a decreased both the number of infectious viruses secreted, and cell-associated viruses. Together the results provide good arguments for a conserved role of Rab27a in cytomegalovirus production in several species.

## Discussion

HCMV assembly requires spatiotemporally controlled interaction of viral proteins, with largely unknown cellular factors, within the context of cellular membranes. Once assembled, mature HCMV particles are transported in vesicles and secreted. To gain insight into the mechanisms controlling these processes, we have analysed the role of Rab27a, a key regulator of LRO motility and secretion. HCMV infection increased the expression of Rab27a, and shifted the distribution from scattered cytoplasmic punctae to sites where new HCMV was assembled. Rab27a was associated to virus-wrapping membranes and tubulovesicular structures, and was found to be incorporated into the viral envelope. CMV production was reduced after depletion of Rab27a and in Rab27a-deficient cells. Based on these results, we propose that CMV exploits the cellular machinery involved in LRO function, for its assembly.

LRO are related to secretory granules. Unlike secretory granules however, LRO derive their content mainly from the endocytic pathway [12]. We have shown that HCMV buds into membranes that contained both TGN and endosomal markers [4], [9], [10], that together with the identification of Rab27a as a player in HCMV production support a relationship between HCMV with LRO. HCMV is not the only herpesvirus that interacts with LRO, since human herpesvirus 6 (HHV-6) also buds into membranes that contain both TGN46 and CD63 [28] indicating that HHV-6 and HCMV may use the same source of membranes for their envelopes. In addition, Mori and colleagues suggested that HHV-6 virions exploit MVBs and the exosomal pathway for release. It has recently been shown that Rab27a controls exosome secretion [29]. Our functional studies showed a reduction in the number of infectious secreted viruses, that together with the localisation of Rab27a on vacuoles enclosing virions and DBs strongly suggest the importance of this small GTPase in HCMV secretion. Exosomes play roles in different physiological processes, and hematopoietic cells such as activated cytotoxic T cells secrete exosomes containing lytic effectors [30]. Interestingly, hematopoietic precursor cells harbour latent viruses and virus reactivation occurs in mature monocyte-derived macrophages and dendritic cells which may function as vehicles of virus transmission [31], [32]. It is tempting to speculate that HCMV viral secretion might be regulated and that Rab27a contributes to specific and efficient transmission of virions. Importantly, we found that lack of Rab27a reduces cell-associated infectious viruses production, indicating that Rab27a regulates HCMV morphogenesis. Since CMV production is not completely inhibited even in Rab27a deficient cells, other Rabs may participate in CMV morphogenesis, as suggested by the association of several Rabs on herpes simplex virus type 1 particles [33] and lytic granules of NK cells [34].

Maturation of lytic granules in cytotoxic lymphocytes requires the heterotypic fusion of Rab27a^+^ MVBs with Rab11^+^ recycling endosomes which then translocate together with lytic granules towards the immunological synapse [35]. This sequence of events is remarkably similar to HCMV maturation and production. HCMV uses MVB and recycling endosomes membranes for envelopment [4], [9], [10], [36] and Rab27a is associated to the envelope of viral particles and DBs, while Rab11 is also required for infectious virus production [36]. Together these data suggest that heterotypic fusion of Rab11^+^ membranes with Rab27^+^ MVBs is required for HCMV morphogenesis. Our data also support the notion that HCMV uses multiple cellular trafficking pathways and compartments to assemble infectious virions [37]. These pathways may be redundant and could explain the partial effect observed in CVM assembly/secretion in Rab27a-depleted and -deficient cells.

In conclusion, our study supports a conserved cellular mechanism that underlies assembly of HCMV and maturation of LRO. Understanding the molecular mechanisms at the interface of the virus-host interactions is crucial for the identification of novel targets for future antiviral strategies.
